# Community-based TB testing as an essential part of TB recovery plans in the COVID-19 era

**DOI:** 10.5588/ijtld.21.0077

**Published:** 2021-05-01

**Authors:** N. Zokufa, K. Lebelo, D. Hacking, L. Tabo, P. Runeyi, N. Malabi, S. B. Sibanda, T. Cassidy, G. Makanda, B. Norman, S. Khuzani, J. Furin, C. Jonker, B. Nkasana, V. Scott, C. Pfaff

**Affiliations:** 1 Médecins Sans Frontières, Khayelitsha; 2 Division of Public Health Medicine, School of Public Health and Family Medicine, University of Cape Town, Cape Town, South Africa; 3 Department of Global Health and Social Medicine, Harvard Medical School, Boston, MA, USA; 4 City of Cape Town, Department of Health, Cape Town, South Africa

Dear Editor,

Modelling has suggested that the COVID-19 pandemic may result in over a million excess TB deaths globally, some of which may be due to missed TB diagnoses.^1–5^ In South Africa, a high HIV and TB burden country, the national lockdown in response to COVID-19 was associated with a 59% decline in median daily GeneXpert (Cepheid, Sunnyvale, CA, USA) TB tests, and a 33% decline in the number of TB diagnoses.^6^ This trend has also been observed in Khayelitsha, a low-income, high TB prevalence, peri-urban area in Cape Town. These drop-offs in TB testing persisted even after the initial peak of the first wave of COVID-19. Most South African efforts to improve access to TB diagnosis and treatment services were tied to health care facilities^7^ that were themselves severely affected by COVID-19. Utilization of available services was also impeded by multiple access barriers, including avoidance of health care facilities due to COVID-19 risk.^8^ Building on a history of successful community-based initiatives, Médecins Sans Frontières (MSF) in collaboration with the City of Cape Town launched the “Tuberculosis Neighborhood Expanded Testing” (TB NET) Project in Khayelitsha to improve access to TB testing and treatment. This initiative was based on studies documenting a high yield of neighborhood-based TB screening focused on households in the vicinity of identified index patients.^9^ TB NET target neighborhoods were selected in conjunction with the health facilities based on the addresses of people newly diagnosed with TB within the preceding 3 months, and the population density and size of each neighborhood. Once a target neighborhood was identified, the community leaders were consulted about the campaign. Individuals with TB were not revealed, but the high incidence of TB was emphasized. This was followed by a workshop with community leaders and stakeholders about TB symptoms and diagnosis, including how to produce sputum.

Following consultation, a TB NET campaign was launched in the selected neighborhoods, each lasting 3–4 days. On the first day, MSF health promoters and community leaders went door-to-door in the target area to educate people about TB symptoms and sputum production. Sputum jars were distributed to anyone who was interested in testing themselves or household members. On the final 2–3 days, the team was stationed at pre-selected drop-off points, where community members were able to leave their sputum samples. The number and location of drop-off points was selected in consultation with the community leaders based on the size of the area, safety and convenience. At the end of each day, the sputum samples were taken to the laboratory by the MSF teams. Results were sent to the facilities and captured into routine data systems. Negative results were communicated via SMS. Those that tested positive were called, followed-up and linked into care. We conducted four campaigns in the catchment area of three clinics (Figure, Panel A). Out of approximately 1600 sputum jars distributed, 151 samples were returned and sent to the laboratory. Of these 151 samples, 140 were tested successfully, and 12 (7.9%) tested positive, one of which was rifampicin-resistant (for results by site see Figure Panel B). All 12 (100%) individuals who tested positive for TB were successfully linked to care — attending the clinic and initiating treatment. Of the 12, 9 were new positives, and 3 had previously been treated.

**Figure 1.**
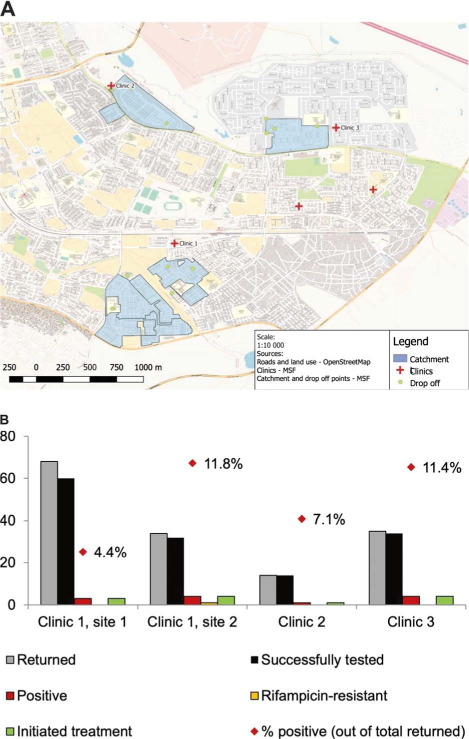
A) Map of target areas and drop off points; B) results of outreach TB testing by site.

We have demonstrated the feasibility of a community-based, neighborhood-focused screening model that shows promising yield and linkage outcomes. Community screening for TB frequently involves screening for symptoms and then referring people to facilities for further testing.^10–12^ In our pilot, we focused on allowing people to self-identify as possibly having TB. We also prioritized convenience by providing sputum jars and allowing people to drop-off sputum samples at a community site. We encouraged participation by educating people about TB symptoms, but not requiring referrals based on symptoms before testing. Nevertheless, we achieved a yield of 7.9% positivity of those tested, higher than other community-based testing.^10,13,14^ This higher rate of positivity could be due to a variety of factors that merit exploration in further studies, including targeting high-risk neighborhoods, engaging with community leaders, utilizing skilled health promotion teams, and allowing individuals to identify their own TB symptoms and risk. It could also be because there was more TB circulating in the community due to the impact of COVID-19. Linkage to care from community screening for TB (and HIV) is often a challenge.^10,12,15^ Once confirmed positive, 100% of people in our pilot attended the clinic to initiate TB treatment, possibly due to the enhanced linkage support provided by the TB NET team. These successes may be explained by the health promotion and community engagement which were key to this intervention, as well as the reduction in clinic interactions required between diagnosis and treatment initiation.

Despite these successes, this intervention faced several challenges. There were a few instances of households refusing TB education because of stigma around the disease. Sputum was not successfully produced in 11 samples (7%), so could not be tested. Finally, although approximately 1400 sputum jars were distributed, only 151 were returned. Nonetheless, we considered broad, unconditional distribution of sputum jars to be a low-cost method of covering a large number of people without requiring them to report symptoms.

The COVID-19 epidemic has had a devastating impact on TB services, with a predicted excess 6.5 million people developing active TB disease by 2025.^1^ Innovative models of TB testing are urgently needed to respond to this crisis. In the context of the ongoing COVID-19 epidemic, in an area with a high TB burden, our community screening model provided an alternative to clinic-based screening and sputum collection. This may be safer and less burdensome for people and clinicians. The high positivity yield of our pilot also suggests that the response to COVID-19 has led to missed TB diagnoses. Although COVID-19 has cast a shadow on TB elimination efforts, it has also forced the community to adapt and develop more community-friendly and person-centered approaches such as TB-NET. If implemented more widely, efforts such as this could turn what appears to be TB’s darkest hour into its finest moment.
